# Haptic, Virtual Interaction and Motor Imagery: Entertainment Tools and Psychophysiological Testing

**DOI:** 10.3390/s16030394

**Published:** 2016-03-18

**Authors:** Sara Invitto, Chiara Faggiano, Silvia Sammarco, Valerio De Luca, Lucio T. De Paolis

**Affiliations:** 1 Human Anatomy and Neuroscience Laboratory, Department of Biological and Environmental Science and Technologies, University of Salento, Campus Ecotekne, Via per Monteroni, Lecce 73100, Italy; 2 University of Salento, Campus Ecotekne, Via per Monteroni, Lecce 73100, Italy; chiarafaggiano0@gmail.com (C.F.); silvia.sammarco@yahoo.it (S.S.); 3 Augmented and Virtual Reality Laboratory (AVR Lab), Department of Engineering for Innovation, University of Salento, Campus Ecotekne, Via per Monteroni, Lecce 73100, Italy; valerio.deluca@unisalento.it (V.D.L.); lucio.depaolis@unisalento.it (L.T.D.P.)

**Keywords:** virtual training, event-related potentials, interactive entertainment

## Abstract

In this work, the perception of affordances was analysed in terms of cognitive neuroscience during an interactive experience in a virtual reality environment. In particular, we chose a virtual reality scenario based on the Leap Motion controller: this sensor device captures the movements of the user’s hand and fingers, which are reproduced on a computer screen by the proper software applications. For our experiment, we employed a sample of 10 subjects matched by age and sex and chosen among university students. The subjects took part in motor imagery training and immersive affordance condition (a virtual training with Leap Motion and a haptic training with real objects). After each training sessions the subject performed a recognition task, in order to investigate event-related potential (ERP) components. The results revealed significant differences in the attentional components during the Leap Motion training. During Leap Motion session, latencies increased in the occipital lobes, which are entrusted to visual sensory; in contrast, latencies decreased in the frontal lobe, where the brain is mainly activated for attention and action planning.

## 1. Entertainment, Virtual Environment and Affordance

Entertainment is becoming an extremely strong field from an economic point of view, so as to arouse interest among economists, such that the modern age is taking the name of “entertainment age” [1], both from the engineering and psychological research points of view. Recently, in fact, some studies developed a new field of research, called “Psychology of Entertainment”, which takes the playful processing and its relationship into consideration with learning, perception, emotions, and communication, through an interdisciplinary approach also useful to marketing, to communication sciences, and cognitive and clinical neuroscience (e.g., the development of serious therapeutics games [2]).

Until now, this research has been analyzed simply through behavioral analysis (e.g., the study of role playing in learning levels and behavioral reaction times), but it becomes more and more evident the need to investigate the effects of the game through psychophysiological variables. This is also due to the fact that evaluating entertainment technology is challenging because success is not defined in terms of productivity and performance, then compared to objective categorization, but in terms of enjoyment and interaction which, instead, are subjective categorizations, strongly related to both a perceptual approach and an emotional approach [3,4].

Around this theme, we are developing various studies in order to analyze and implement haptic interaction aspects; for example, in an interface built from the Disney Group, is looking to increase an augmented reality product through tactile stimulation [5].

To investigate these aspects we aim to understand the properties of an interactive space and activations that can be produced by objects that lead to actions, so that this analysis allows us to better understand the different possible uses of entertainment technology, as well as ergonomic implementations that we can improve (e.g., studying how the perceptual levels process the virtual product). To do this we introduce an extremely important concept for cognitive science: the concept of affordances. Gibson [6] was the psychologist who introduced the affordance theory: he highlighted that the dynamic pattern of the optic flow can reactively lead the interaction in the environment. He introduced the term “affordance” to give an active meaning to the visual perception of the environment: according to his new theory, such perception directly includes the potential actions that a perceiver can carry out without activating high-level reasoning processes about object properties [7]. The affordance is directly perceivable by the organism because there is information in the environment that uniquely specifies that affordance for this organism. In other words, Gibson’s affordances introduce the idea of the actor-environment mutuality: the actor and the environment make an inseparable pair. This idea was different from the contemporary view of the time that the meaning of objects was created internally with further “mental calculation” of the otherwise meaningless perceptual data. Indeed, Gibson’s work was focused on direct perception, a form of perception that does not require mediation or internal processing by an actor [8,9].

From a manipulation perspective, for instance, a person watching an object would directly perceive also the object’s “graspability”, “liftability”, *etc.*, as well as shapes and colors [10].

To be graspable, an object must have opposite surfaces separated by a distance less than the span of the hand. In addition to the object itself, the embodiment (in particular the actuators) of the observing agent conditions the object affordance, too. A bottle, for instance, affords grasping for human beings, whereas it might afford a biting action for dogs, and another different action for ants. Furthermore, the affordance is perceived visually: the surface properties are seen relative to the body surfaces, the self, they constitute a seat and have meaning. The size of an object that constitutes a graspable size is specified in the optic array, and if this is true, it is not true that a haptic sensation of size has to become associated with the visual sensation of size in order for the affordance to be perceived. A five-inch cube, for example, can be grasped, but a ten-inch cube cannot [11]. The affordance concept assumes that the resulting sensorimotor processing tends to trigger or prepare an action automatically in a reactive pattern (even though this tendency can be significantly influenced by the agent context and goals).

Recently, the Gibsonian affordance concept has been expanded to include also the brain representations of affordances (*i.e.*, the possible sensorimotor interactions linked to objects; see for instance recent papers on computational models of affordances [12,13].

They worked on memory span, involving objects with handles oriented to the left or right side. These representations include both the features involved during a handling object, such as the object size and location, and the relation between the object and the agent’s body, such as the proximity or the contact between the object and a distal effector (hand).

Nowadays Gibson’s ecological framework is considered a valid functional approach for defining the level of realism of experience in the design of virtual environments [14]. For instance, the perception of affordances could potentially improve the sensorimotor assessment of physical presence, which is the sensation of being physically located in a virtual world [15]. In this context, Lepecq [15] studied the behavior of some subjects walking through a virtual aperture of variable widths. The analysis assumed the sense of presence leads the subjects to change their body orientation according to the aperture width during the walk: this suggested a significant similarity between the locomotor postural patterns of subjects walking through a virtual aperture and those of subjects walking through a real aperture [16]. A behavioral transition from frontal walking to body rotation was observed in most subjects when the virtual aperture narrowed.

Finally, researchers proposed a conceptual model representing affordances’ arousal in virtual environments (VEs) through sensory-stimuli substitution. Such a model can provide VE designers with several useful hints for conceiving more ecologically valid environments [14]. According to this literature, there is a need to integrate a comprehensive theory of perception into VE design. Theories of direct perception, in particular affordance theory, may prove particularly relevant to VE system design because affordance theory provides an explanation of the interaction of an organism with its environment [14].

Some recent works analyzed the cognitive process in the human brain in terms of event-related potentials (ERP) during virtual reality experiences. An event-related potential measures the brain response to a specific sensory, perceptual, cognitive, or motor event [17]. For these reasons, realistic and immersive environments provided by virtual reality technologies can be used to setup reliable experimental scenarios to analyze the cognitive process mechanism of the human brain.

In this regard, some studies have already tried to shed light on factors that affect the efficient and rapid acquisition of knowledge using this technology and demonstrated that the right cerebral hemisphere appears to be more activated than the left during navigational learning in a VE. These results underlined the implications of the use of VEs for training purposes and many assist in linking the processes involved in navigation to a more general framework of visual-spatial processing and mental imagery [18].

A recent experimental work [19] focused on a virtual reality traffic environment to study the cognitive processing in the human brain in terms of event-related potentials: for this purpose, traffic signs are showed during the experiment with correct and incorrect background colors. The results revealed that the human brain can reply more quickly in presence of simpler contents and stronger color contrast between the background and the foreground. Indeed, according to this approach, some researchers consider augmented reality as a proper instrument to achieve a better adherence to the right procedures thanks to its ability to increase users’ motivation [20].

This paper analyses the perception of affordances in a virtual environment in terms of psychophysiological and behavioral parameters, especially using event-related potentials components, according to the neuroscientific aspect of cognition. For our empirical study, we chose as experimental scenario the interaction with a virtual reality application through the Leap Motion controller [21], a small and low-cost gesture detection system [22] designed for interactive entertainment. The software applications provided with this device reproduce the proper interactive experience for our study about the perception of affordance.

In accordance with our model, the adoption of new information and communication technologies within the education field is allowing the development of several interactive systems and software applications. Their technological novelty makes the interaction with them even more interesting, compelling and fascinating. Their adoption allows the user to play, to entertain and to memorize some learning objects more easily and with more involvement. They have expanded the possibilities for experimentation within fields and situations that can be simulated by virtual environments.

### 1.1. Gestural Technologies

Gestures [22] are new forms of communication based on the association of particular messages and meaningful commands with well-defined positions or movements of some parts of the human body. They typically deal with finger and hand movements, but some authors are investigating also the possibility to track head and eye movements and face expressions [23] by exploiting the proper face tracking API provided by devices’ vendors [24].

Gestures represent a more natural form of human-computer interaction than traditional devices such as mouse and keyboard: indeed, they mostly derive from body language, which is part of the natural communication among people. In gesture-based environments, users can pay more attention to the output on the screen, since they do not have to look at the input devices anymore.

Moreover, gestures are a more practical alternative to voice-recognition systems, which require a long and complex training phase to adjust to the voice tone and the user’s diction.

In the last years a lot of devices and systems have been designed for gesture detection by engineers and software developers. However, hints provided by human-interaction experts should be taken into account in the design process to make gestural technologies more usable and consistent with natural interaction forms. Moreover, the distinction between real control gestures and accidental movements is still an open problem.

In the earlier interaction systems based on hand gestures users had to wear electronic gloves [25,26] containing several sensors. The first systems could only detect static hand postures, sometimes coupled with position and orientation in the space [25]. Then, in a later phase, some more advanced systems introduced dynamic gesture detection [27], which also include movements, and finger tracking [28].

An important drawback coming from the adoption of electronic gloves is related to the calibration of those systems, whose parameters need to be tuned according to hand geometry [29].

Some other gesture detection systems [30,31] apply color segmentation on colored gloves worn by users or use vision-tracked fiducial markers [32] placed on some handheld devices [33] or even directly over hands and fingertips [34].

Another wearable device is a wristband [35] based on the electromyography technique, which consists in retrieving signals from the electric potential of human muscles.

Wearable devices are often cumbersome and may limit hand movements due to the presence of sensors and wires. The consequent constraints on the degree of freedom of movements partially reduce the range of users’ gestures. Moreover, the general user experience may be negatively affected. Users often do not perceive this form of interaction as being as natural as that offered by vision-based systems.

Interactive tabletop devices can detect gestures by exploiting histogram methods [36] or some more expensive touch-sensitive surfaces [37], but they require the hand to keep a fixed posture.

The vision-based recognition system described in [38] uses a simple webcam to acquire images of the user’s hand and recognize Indian signs (representing numbers from 0 to 9).

The main drawback of camera-based systems is related to their restricted interaction area, which is limited to a specific field of view near the device. Furthermore, lenses or sensors issues, lighting problems, and objects in the scene foreground/background could significantly affect their accuracy and reliability [22]. Some systems try to overcome these problems by exploiting electric fields [39] or Wi-Fi signals [40,41,42]: they recognize gestures by detecting the disturbance produced by body parts.

Nevertheless, nowadays the most common gestural technologies, introduced by videogame and entertainment companies (e.g., Microsoft, Nintendo, and Sony), rely either on handheld devices or on cameras performing motion tracking.

**Figure 1:**
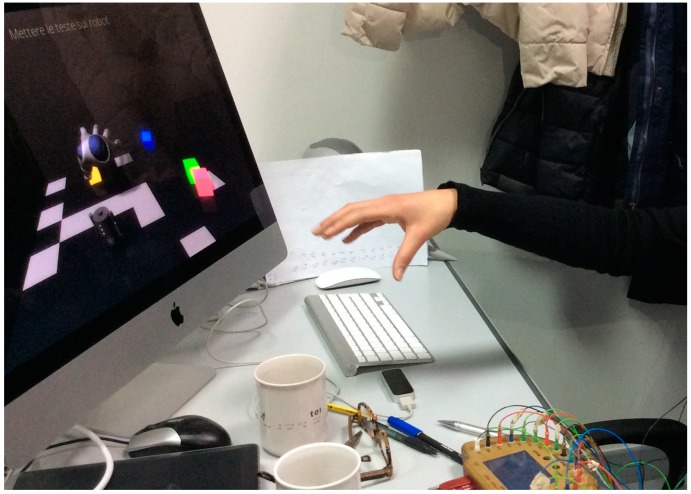
The use of Leap Motion controller during the experiments.

The Leap Motion controller [21] is a small, easy to-use, and low-cost device designed to capture the movements of human hands and fingers. Figure 1 shows the use of the Leap Motion during the experiments.

The Sony PlayStation Move controller [43] detects movements in the 3D space thanks to a multi-colored light source and a webcam.

The Nintendo Wii remote controller (Wiimote) [44] is equipped with accelerometers, infrared detectors, and a LED sensor bar. The MotionPlus add-on can detect change in orientation along the three axes thanks to some inertial gyroscopes.

A comparison between Leap Motion and Wiimote in terms of accuracy and tracking reliability can be found in [45]. The Wiimote is more reliable in movement detection than the Leap Motion controller, which is not able to detect twisting motion properly; on the other hand, the Leap Motion is able to detect finger gestures, such as circular movements and forward/downward movements. Furthermore, the Leap Motion controller allows a more natural form of interaction: users do not have to hold any particular object and, thus, can freely move their hands and fingers in the space.

Unlike Sony and Nintendo, Microsoft introduced Kinect [46], a markerless webcam-based system that does not need any handheld device. In fact, two different Kinect versions were released, characterized by different architectures and performance [47].

Among all the presented devices, we chose the Leap Motion controller for the experiments described in this paper mainly due to its usability: the user can move freely his hand within the interaction volume without any need for grasping or wearing devices. Moreover, the Leap Motion may give a higher sense of realism due to a more accurate gesture detection compared to other devices such as the Kinect. Indeed, although in a combined use the detailed information in Kinect’s full-depth map could overcome some limitations of the Leap Motion [48], even the second Kinect version may fail in properly detecting hands and fingers, especially during abrupt movements [49].

### 1.2. EEG, Virtual Interaction and Motor Imagery

This approach is performed within a paradigm of cognitive neuroscience, specifically within the studies of motor affordances. This paradigm is developed through the technique of electroencephalography (EEG) and, in particular, event-related potentials (ERPs). EEG is a procedure that measures neural activity of the brain through electrodes placed on the scalp. The EEG is the result of ongoing activity of numerous neurons under the scalp, so, it is very improbable to see a single-peak evoked response in a single task. To obtain a clear wave elicited by the task, the trials must be repeated [50].

Moreover, in psychophysiology, go-no go tests are employed to measure subject attentional and decisional processing. For example, a go-no go task requires one to perform an action given certain stimuli (e.g., press a button where the stimulus is recognized) and inhibit that action under a different set of stimuli (e.g., not press that same button, where the stimulus is not recognized).

Specifically, in relation to this perspective, we started to study, according to a survey of psychophysiological entertainment interface, the possibilities for differentiation of this with a real or imagery interface [3], and the analysis of the difference between a real haptic manipulation, built with a 3D printer, and an augmented manipulation according to learning styles [51].

In a previous study [51], Invitto, *et al.* investigated through an “augmented game”, Advanced Distributed Learning (ADL). ADL is a kind of learning mediated by new technologies. The ADL also makes use of augmented reality, which takes place through processes of virtual manipulation.

The experimental research focused on an augmented reality product (Dune AURASMA), and 3D objects printed with a 3D scan. We analyzed the possibilities of interaction and manipulation of shapes in augmented reality and in a real context. Literature shows that there are different modules within the occipitotemporal cortex that receive both visual and somatosensory inputs and it explains how these can be integrated in the learning process.

These cortical modules can be activated in the evaluation of the various aspects of surface properties of objects, such as the 3D shape, as well as in visual and haptic movements. The work analyzed ERP components variations during two different kinds of learning training: the same objects are manipulated either in augmented reality or during the condition of real haptic manipulation and the variations due to different learning styles are investigated.

We considered four scales of learning style: visual verbal, visual non-verbal, kinesthetic, and analytical. The subjects performed a training lasting 5 min consisting of haptic manipulation of 3D models, obtained through modeling in 3D Blender 2.74 and manipulation in augmented reality, presented through Dune^®^ Aurasma models. After each training the subjects had to perform a recognition task of the same stimuli (presented in 2D), during an EEG recording. A general linear model was computed to investigate the research hypothesis. Results of this study highlighted an effect on ERPs components due to learning styles. The subjects with high scores of visual non-verbal learning style showed higher amplitude in the central, occipital, and parietal channels in early components (appointed to attentional processing) of ERPs. Meanwhile, the subjects with visual verbal learning presented higher amplitude, in general, in the cognitive component. According to this result, we can conclude that learning styles are involved in perceptual levels during an augmented training and according to the prominent style, processing involves different ERP components and different brain areas. The learning style affects more these variations when the mode of training is through augmented reality, where the visuomotor process is prevalent.

In this work, with respect to our previous studies, we implemented and used the concept of “motor imagery” to convey the idea of a mental interaction built on the sensorimotor mental imagery.

The aim of the paper is to investigate how we can use tools designed for entertainment, in a useful way in learning processes and interaction. Using electrophysiology techniques and, in particular, the event related potentials, in this study we investigated cortical responses and attentional arousing during the interaction with the interactive game Leap Motion, rather than when a subject manipulated a real or imagined object with motor affordance. This allows us, in this case, to understand whether these new gaming tools, which seem to be a middle ground between the game “acted” and the play “imagined” cannot be only new playful instruments, but also become the means that allow and facilitate learning.

## 2. Method

### 2.1. Participants

Our sample was composed of 10 university students matched by age and sex (five men and five women). The sample of recruited volunteers had normal hearing, normal or corrected-to-normal vision, and a right manual dominance. The subjects recruited had no previous experience of EEG and cognitive tasks. The subjects performed in the baseline condition study (motor imagery training) and in the immersive affordance condition (a virtual training with Leap Motion and a haptic training). None of them had previously taken part in experiments like these. All participants gave written informed consent according to the Helsinki Declaration.

### 2.2. Measurements and Stimuli

After reading the informed consent form and task description, participants completed the questionnaire with anagraphical data.

Subjects had to perform three training sessions: a motor imagery training, a haptic object manipulation training, and Leap Motion manipulation training. In the three training sessions, subjects were shown objects with affordance of grasping: a cup, glasses, scissors, pot handle, a computer mouse, a fork, a pen.

After each training the subject had to perform a go-no go task, during an EEG recognition task recorded through E-Prime 2.0 presentation (Psychology Software Tools, Inc., Sharpsburg, PA, USA).

#### EEG

During the images presentation task we recorded a 16 channel EEG using Brain AMP—Brain Vision Recorder. We considered the event-related potential (ERP) for grasping objects.

During the computer-supported attention task, EEG was recorded by 16 surface recording electrodes, belonging to the Brain Vision Recorder apparatus (Brain Products, GmbH, Dusserdolf, Germany). A further electrode was positioned above the right eyebrow for electro-oculogram (EOG) recording. The ERP’s analysis was obtained through the Brain Vision Analyzer. Time offline analysis was from 100 ms pre-stimulus to 500 ms post-stimulus with 100–0 baseline correction.

Thereafter, trials with blinks and eye movements were rejected on the basis of a horizontal electro-oculogram with an ICA component analysis. An artifact rejection criterion of 60 V was used at all other scalp sites to reject trials with excessive EMG or other transient noise. The sampling rate was 256 Hz. After a transformation and re-segmentation of data with the Brain Vision Analyzer, the artifact-free EEG tracks corresponding to the affordance object, marked by the motor response, were averaged in each case to extract the main waveforms, the N1 in the 120–175 ms time range, the P1 in the 175–250 ms time range, and the P3 component in the 260–400 ms time interval, according to the literature. We performed a semi-automatic peak detection with the mean of the maximum area for the different components of the ERP waves.

### 2.3. Procedure and Task

**Figure 2:**
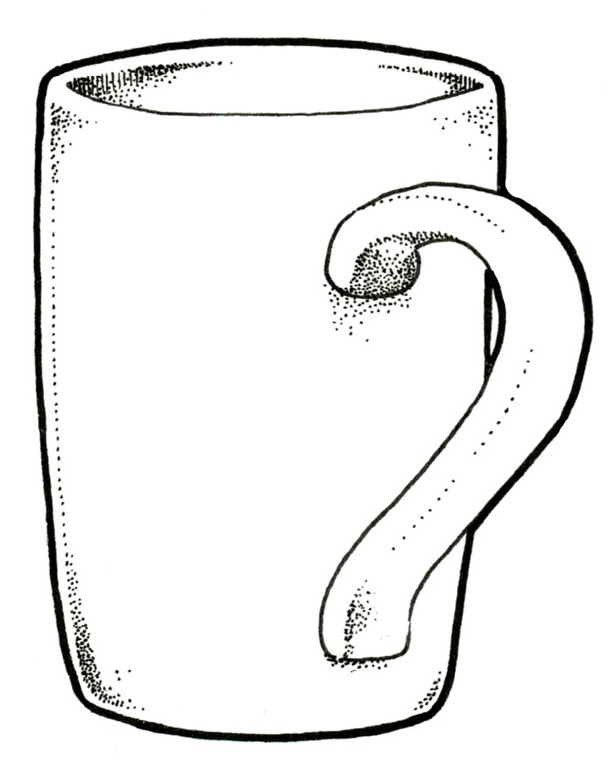
Example of an object with grasping affordance.

Our study/experiment consisted of an analysis of affordances perception in VR through an electrophysiological variable (ERP). The task in baseline condition shows, in a pseudo-random way, images (pictures in 2D presentation, with the E-Prime 2.0 software) such as colored frames, no grasping objects (e.g., table, chair), and grasping objects (e.g., glasses, cup, scissors, mouse, pen, and fork, e.g., Figure 2). The grasping objects were presented with a percentage of 20%.

**Figure 3:**
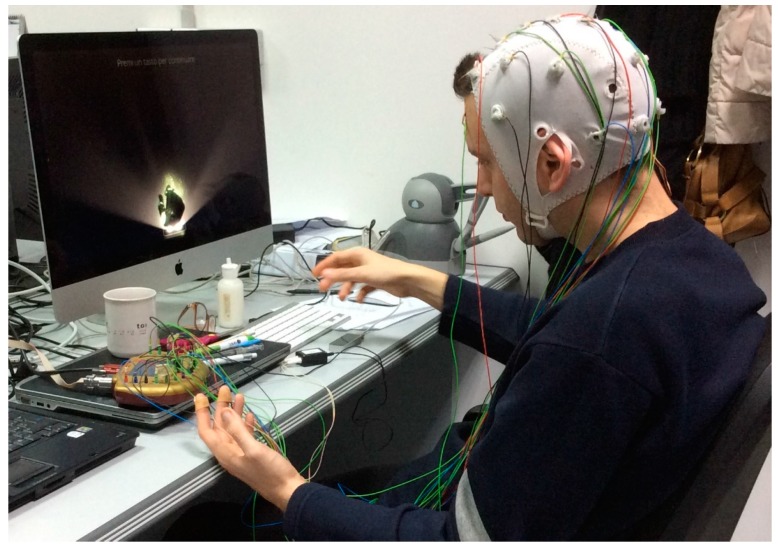
Subject during virtual training with Leap Motion.

Subjects were seated in a comfortable chair 100 cm from the display monitor; their midsagittal plane was aligned with the center of the screen and with the center of the Leap Motion. Subjects performed three different tasks during the experiment (Figure 3):

- Task Training A, in which they were asked to think using the objects with affordance of grasping (the objects were positioned in front of the subject, on the table);
- Task Training B, in which they were asked to think using the objects while interacting with the Leap Motion Playground app (they had a visual feedback of the hand motion on the screen);
- Task Training C, in which they really used the grasping objects.

Each training task had a duration of 2 min.

The training were not presented in sequence but follow a randomly alternating order for each subject.

After each training session, the subject had to perform an E-Prime experiment in which he had to recognize, among various objects and colored frames, the objects he had previously seen during the training. The triggers of affordance images were used for ERP segmentation analysis.

The recognition task images were selected through a repertoire of neutral images (colored squares on a light background), non-target images (animals, everyday objects), and target images (the same grasping objects used in the previous training session).

**Figure 4:**
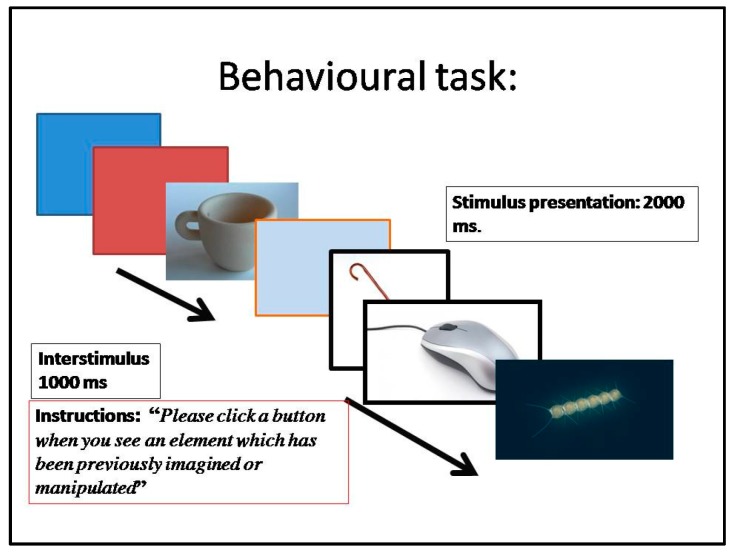
Behavioral task: go-no go task.

All stimuli had dimensions of 240 × 210 pixels, and were displayed centrally on a light grey background and to the same level of brightness on the computer monitor. The task was administered via E-Prime software 2.0. The task paradigm was a go-no go presentation (Figure 4). Each trial lasted 600 s, with a stimulus duration of 2000 ms and 1000 ms of interstimulus duration.

The participants were instructed to stand upright with *ca*. 75 cm between the front edge of the chair and the floor of the computer screen. The following instruction message was shown to each user: “Please click a button when you see an element which has been previously imagined or manipulated.”

## 3. Statistical Analysis and Results

A statistical ANOVA method was performed to analyze behavioral and electrophysiological data, using the three condition of trainings as independent variables, and reaction time (behavioral value) and wave’s component (psychophysiological value) as dependent variables.

### 3.1. Behavioral Task Analysis

The result of reaction time is significant (F = 4.009; *p* = 0.020); *post hoc* analysis shows a significant difference (*p* = 0.016) between condition 2 and condition 3 (Leap Motion training and real training in the direction of slower reaction time in the Leap Motion condition (mean of 1.254 ms) *versus* haptic training (mean of 934 ms). There was no significant effect on reaction time in the motor imagery condition (mean 1.112 ms).

### 3.2. ERP Analysis

We performed a one-way ANOVA analysis with the training condition as factor (1: motor imagery training; 2: Leap Motion training; 3 haptic training) and latencies and amplitude of N1, P1, and P3 ERP components as dependent variables in the affordance condition.

Main results for N1 waves are reported in Table 1: N1 shows significant values for O1 Latency (F = 5.373; *p* = 0.012), O2 Latency (F = 5.570; *p* = 0.010), and Fz Latency (F = 5.206; *p* = 0.013).

**Figure 5:**
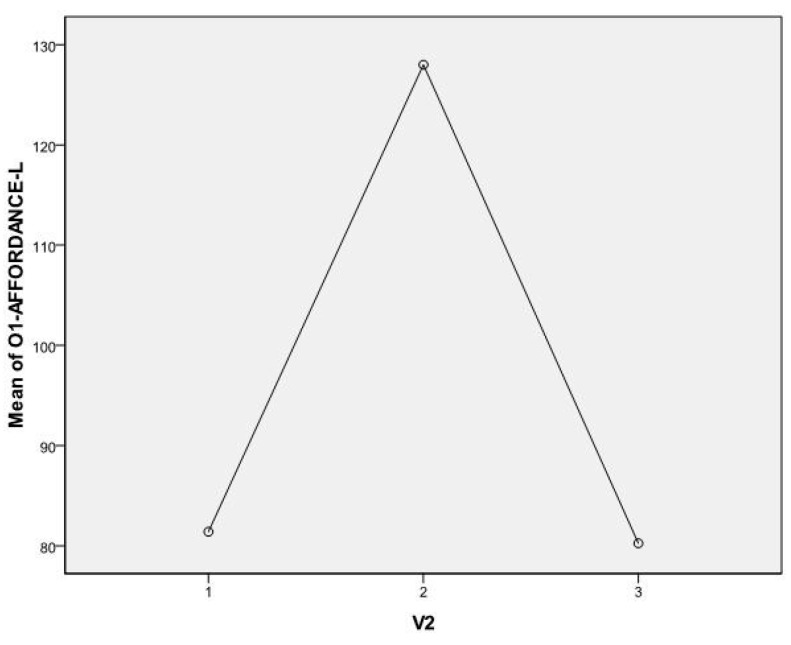
N1 ERP component latency in occipital left lobe: condition 1 (motor imagery), condition 2 (Leap Motion), and condition 3 (haptic manipulation). In the Leap Motion condition N1 latency is slower.

*Post hoc* analysis (Bonferroni test) shows in O1 a significant difference between condition 1 (motor imagery training) and condition 2 (Leap Motion training) with a significant value (*p* = 0.025) and a significant difference between condition 2 (Leap Motion training) and condition 3 (haptic training) (*p* = 0.030). This trend is highlighted in Figure 5. The session after the Leap Motion training presents slower latency in occipital left channel.

**Figure 6:**
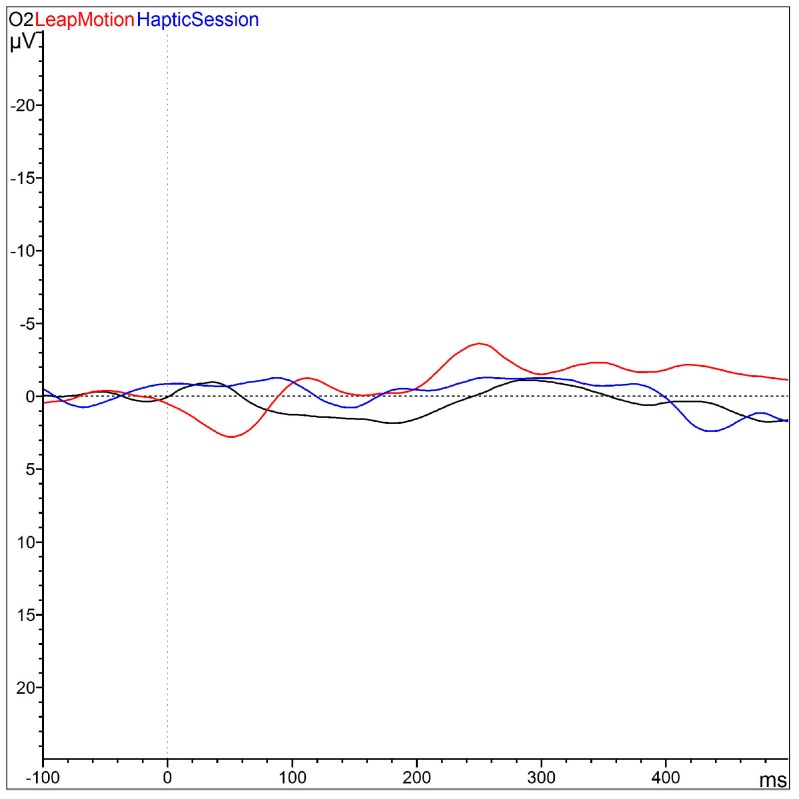
Grand average-matching ERP: black line represents the response of the task after motor imagery training; the red line represents the response after the task of Leap Motion training, and the blue line represents the response after haptic training.

Same trend is present for the O2 latency, where the Bonferroni test indicates a significant difference between 1 and 2 (*p* = 0.044) and 2 and 3 (*p* = 0.015) (Figure 6). Grand average is a method for comparing variability in ERP across subjects and conditions [52].

**Figure 7:**
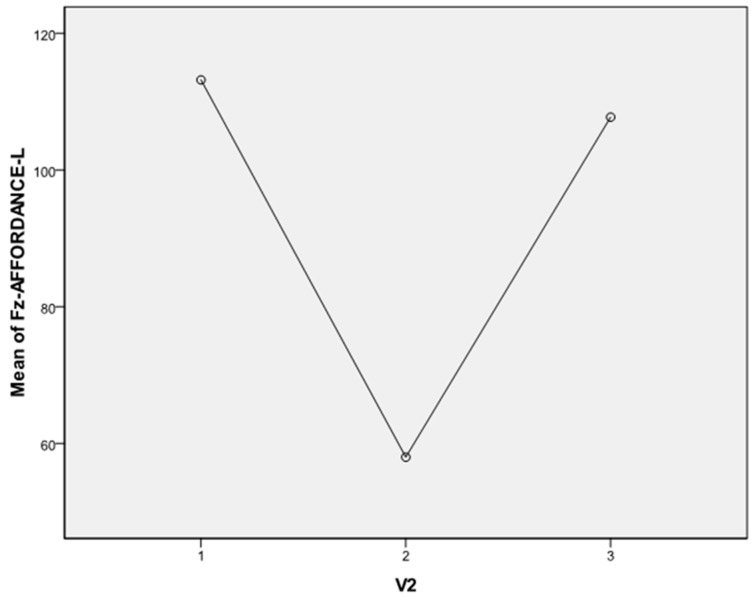
N1 ERP component latency in frontal lobe: condition 1 (motor imagery), condition 2 (Leap Motion), and condition 3 (haptic manipulation); in the Leap Motion condition N1 latency is faster.

**Figure 8:**
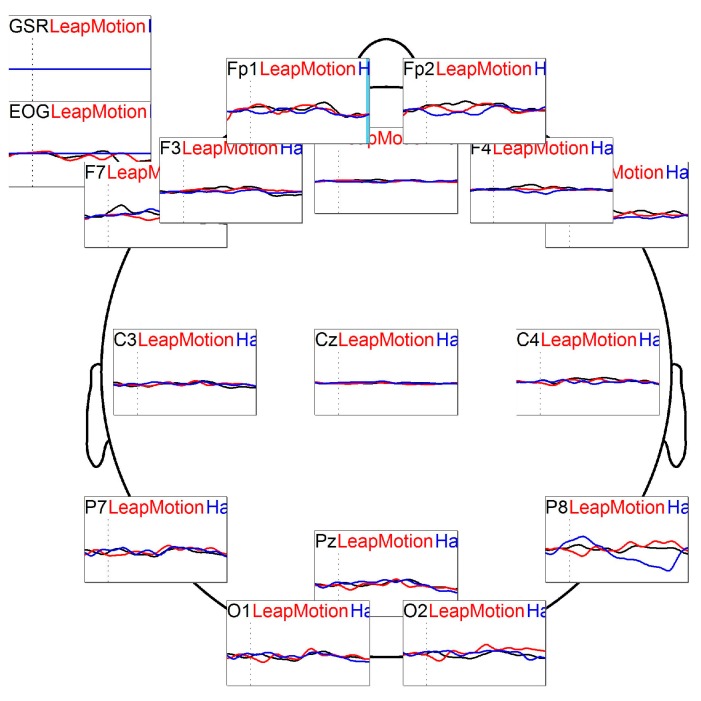
Grand average-matching ERP: black line represents the response of the task after motor imagery training; the red line represents the response after the task of leap motion training, and the blue line represents the response after haptic training. This matching is the ERP trend of the three conditions during the task in all channels recorded.

In Fz latency, the Bonferroni test shows a significant difference between 1 and 2 (*p* = 0.019) and 2 and 3 (*p* = 0.053) as shown in Figure 7. These differences are in direction of a faster latency in the Leap Motion condition. Trend electrodes comparison (grand averages) is showed in Figure 8.

**Figure 9:**
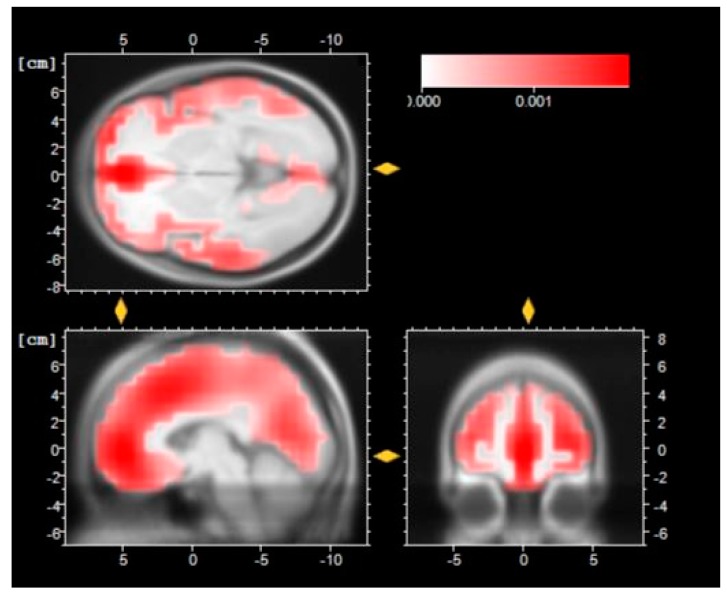
LORETA reconstruction of the N1 component after the Motor Imagery session.

**Figure 10:**
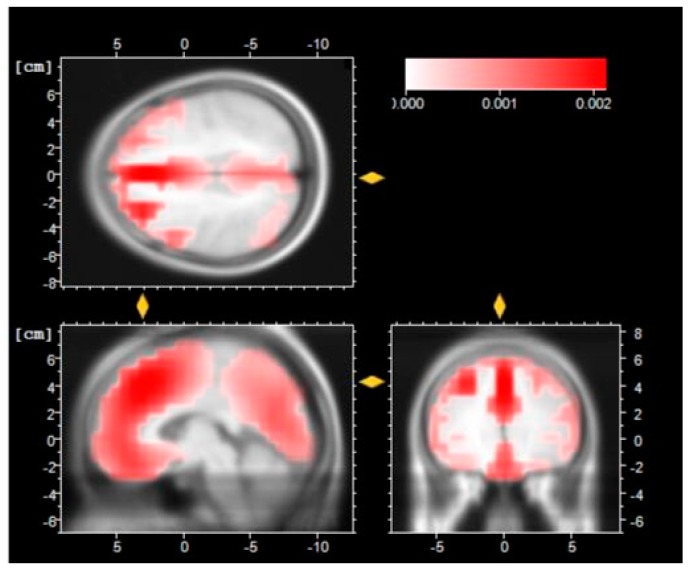
LORETA reconstruction of the N1 component after the Leap Motion session.

**Figure 11:**
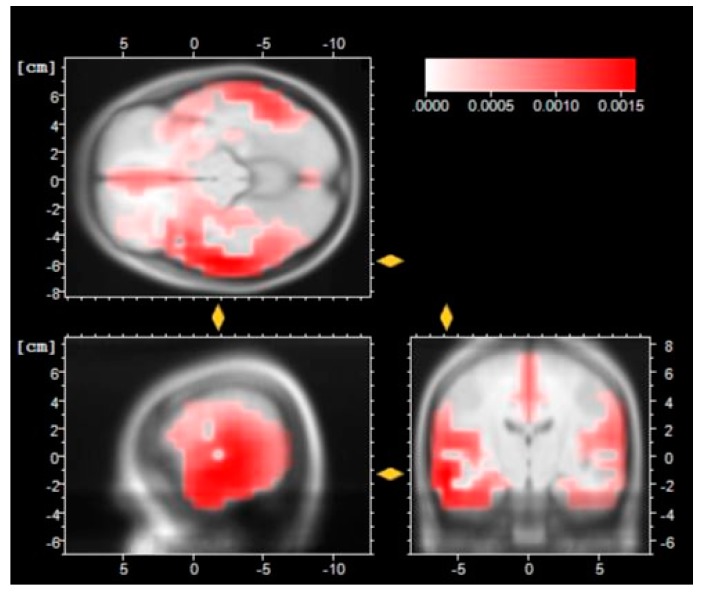
LORETA reconstruction of N1 after the haptic manipulation session.

The low resolution of brain electromagnetic tomography (LORETA [53]) is shown in Figure 9, Figure 10 and Figure 11.

Main results for P1 waves are:

- P1 shows significant value for F4 Latency (F = 4.334; *p* = 0.025);
- *post hoc* analysis (Bonferroni test) shows a significant difference between condition 1 and condition 3 (*p* = 0.031);
- no significant difference in P3.

The LORETA technique is a method that allows to understand the source of electrical activity in the brain based on scalp potentials during an EEG recording. In Figure 9 there is a LORETA reconstruction of the N1 component after the motor imagery task. The reconstruction suggests that there is a deeper cortical level in the frontal lobe, especially in Broadmann Area 10, that is involved in strategic processes of memory and in executive function.

The LORETA recostruction in Figure 10 suggests that there is a deeper cortical level in the frontal lobe, especially in Broadmann Area 8, which is involved when a subject is in the management of uncertainty.

The LORETA reconstruction in Figure 11 suggests that there is a deeper source in cortical level in temporal lobe, in particular in Broadmann Area 22, which is involved in language and prosody processing. These aspects will be discussed in more detail in the next session within the results discussion and conclusion.

## 4. Discussion

The results of this work are sensitive to training conditions; in particular to the virtual Leap Motion training. In the behavioral task we had a significantly slower reaction time in the Leap Motion condition than in the real condition. In the event-related potential result, we found significant values in early components (N1 and P1).

For N1 waves, we had a slower latency in occipital channels and faster latency in frontal channels for the Leap Motion, while we found differences in P1 for real training and motor imagery training sessions with a shorter latency in the frontal right channel only for the motor imagery training condition. There were no results for ERP amplitude and no significant results in the P3 component. Trying to fillet the ERP results and source areas highlighted by LORETA technique, we can see how, after several trainings, different brain sources are involved, notwithstanding the ERPs that are always recorded on the same task. In fact, in our case, this does not change the recognition of the stimulus test, but only the training that precedes this recognition. In the motor imagery task, we see the involvement of the Broadmann area 10 linked to executive functions. This specific part of the cortex is the rostral prefrontal cortex, and is called the gateway of imagination and of executive processing [54]. This is evident if we consider that motor imagery is equivalent in some way to an action planning. It is interesting to see, then as during the use of Leap Motion there is an activation of the dorsolateral prefrontal cortex, which is involved when the subject has to manage a state of uncertainty. From these results, which are also supported by retarded behavioral reaction times of and slowest latencies in the event related potentials, we can say that the interaction with augmented reality, in this case with the Leap Motion, did not directly facilitate the perceptual processes, but it creates a sort of “dissonance”, probably due to an incomplete interaction of sensory systems. Finally the haptic interaction processes, with real objects and in a real environment, using objects with grasping affordances, activate a process in the temporal area, where the haptic interaction and the object visual recognition is strongly related to multisensory processing [55,56].

## 5. Conclusions

In recent years, affordances have attracted considerable interest in the field of cognitive neuroscience. Starting from a more general idea that objects in the environment invite us to act [6] the cognitive researchers now investigate most specific components of actions evoked by objects, as well as the neural correlates of visual-motor associations formed during the experience with them [57].

In this work, we have studied the affordances presenting individual objects with grasping affordance, asking the subject to interact with the objects at different levels. The objects, for example, can be manipulated concretely in a training of haptic handling which, in a virtual way by using the Leap Motion or in an imagination session by means of motor imagery.

After each training session, the subject was subjected to a recognition task of the stimuli, during an EEG recording, which allows us to consider how the brain activations vary depending on the proposed training. Among the ERP’s components we chose the most sensitive to these conditions. N1 is a component of ERP that occurs between 50 and 150 ms and is considered too short to be influenced by top-down influences from the prefrontal cortex.

Some researches show that sensory input is processed by the occipital cortex in 56 ms and that the information is communicated to the dorsolateral frontal cortex where it arrives in about 80 ms [58]. These higher-level areas create the repetition and arousal modulations upon the sensory area processing reflected in this component [59]. Another top-down influence upon N1 was suggested to be efference copies from a person’s intended movements so that the stimulation deriving from them is not processed [60]. Instead, P1 is very sensitive to attentional processing.

In our results, we found a sensitive variation for latencies in these two components but not in the P3 component. This can happen because the sensorimotor and attentional processes are activated in this experimental model in a very early way, through motor training and through the visual affordance of the objects. In behavioral results, we have a slower reaction time in the Leap Motion condition. This could be due to the virtual motion training, which can be mostly considered as a non-integrated sensory multilevel training, because it involves both the thought feedback and the motor/visual feedback, without providing any tactile feedback. This may, indeed, somehow slow down the processes of motor response.

According to our results, we can say that motion training, applied to interactive entertainment (in this experiment Leap Motion training was used as an interactive entertainment), can significantly change the discrimination and retention ability of the presented stimulus. In our study, the motor imagery training and the haptic training can sometimes be in the same results trend as the virtual training; the Leap Motion session changes this trend. Early attentional components in the occipital lobe, which is entrusted to visual sensory, increase latencies when the subject uses the Leap Motion controller.

In contrast, latencies decrease in the frontal lobe, where the brain is involved in attentional arousal, sensorimotor action, and action planning rather than visual perception. In this study we used grasping objects with affordance motion, because they are easier to try during haptic training and because they had an interesting way of being perceived by cognition, because objects have affordance related to action planning. Moreover, affordance allows one to understand the motion way and the grasping way to manipulate the object.

However, in future experiments we will investigate grasping affordance with lateralized readiness potentials, which are a special form of readiness potential (a general pre-motor potential) [61], very sensitive to affordances over a whole range of conditions, even when there is no intention to act or to prepare a manual motor response [62]. In future experiments, we will use the Leap Motion controller and others game interfaces to analyze the virtual interaction and the connections related pleasure, learning, and cortical response. All of these studies and results could be useful for implementing, in a neurocognitive and ergonomic way, a technological and virtual product, too, by employing a device developed with the purpose of entertainment, as a tool of psychophysiological testing.

## Figures and Tables

**Table 1:** ANOVA results and post hoc analysis with mean of latencies (ms).

**ERP N1**	**MotorImageryLat**	**LeapMotionLat**	**Haptic Lat**	**F**	***p***
Fz	113.30 *	58 *	107.75 *	5.206	0.013
O1	81.40 *	128 *	80.25 *	5.373	0.012
O2	89.40	137.56 *	77.75 *	5.570	0.010
**ERP P1**	**MotorImageryLat**	**LeapMotionLat**	**Haptic Lat**	**F**	***p***
F4	100.20 *	122.50	148.41 *	4.334	0.025

* indicates a significant value for alpha ≤0.05.
